# Long-Term Epigenetic Regulation of *Foxo3* Expression in Neonatal Valproate-Exposed Rat Hippocampus with Sex-Related Differences

**DOI:** 10.3390/ijms25105287

**Published:** 2024-05-13

**Authors:** Eun-Hye Jang, Soon-Ae Kim

**Affiliations:** Department of Pharmacology, School of Medicine, Eulji University, Daejeon 34824, Republic of Korea; dmter12@gmail.com

**Keywords:** autism spectrum disorder, valproic acid, *Foxo3*, *Ascl1*, epigenetics

## Abstract

Perinatal exposure to valproic acid is commonly used for autism spectrum disorder (ASD) animal model development. The inhibition of histone deacetylases by VPA has been proposed to induce epigenetic changes during neurodevelopment, but the specific alterations in genetic expression underlying ASD-like behavioral changes remain unclear. We used qPCR-based gene expression and epigenetics tools and Western blotting in the hippocampi of neonatal valproic acid-exposed animals at 4 weeks of age and conducted the social interaction test to detect behavioral changes. Significant alterations in gene expression were observed in males, particularly concerning mRNA expression of *Foxo3*, which was significantly associated with behavioral changes. Moreover, notable differences were observed in H3K27ac chromatin immunoprecipitation, quantitative PCR (ChIP-qPCR), and methylation-sensitive restriction enzyme-based qPCR targeting the Foxo3 gene promoter region. These findings provide evidence that epigenetically regulated hippocampal *Foxo3* expression may influence social interaction-related behavioral changes. Furthermore, identifying sex-specific gene expression and epigenetic changes in this model may elucidate the sex disparity observed in autism spectrum disorder prevalence.

## 1. Introduction

Autism spectrum disorder (ASD) is a neurodevelopmental disorder characterized by early-appearing social communication deficits associated with diverse genetic and epigenetic factors [1,2]. Various animal models have been used to investigate the underlying biological mechanisms and potential treatments for ASD, despite arguments that the behaviors and characteristics displayed by these models may not fully capture the complexity of ASD in humans. Injecting valproic acid (VPA) into rodents during the perinatal period has been reported to induce behavioral changes similar to ASD [3]. Furthermore, behavioral changes persist across generations in prenatal VPA-exposed animal ASD models [4]. As a kind of histone deacetylase (HDAC) inhibitor, a treatment of VPA is expected to induce epigenetic modifications [5,6]. Social-related behavioral changes have also been reported in the neonatal exposure model, in which VPA is administered immediately after birth, and more social-related behavioral changes have been reported in males [7,8].

A relationship between ASD and autophagy has been proposed with various etiological mechanisms for ASD [9]. Autophagy is a cellular process that involves the recycling and removal of damaged or unnecessary cellular components and is crucial for maintaining cellular homeostasis. *Foxo3* is a transcription factor that regulates the pool of neural stem cells (NSCs) by inducing genes that preserve quiescence, prevent premature differentiation, control oxygen metabolism, and induce autophagy [10,11]. Zhang et al. have reported an impaired autophagy mechanism by examining various autophagy markers in the hippocampus of a VPA-induced ASD animal model [12].

Additionally, when we examined the role of *Foxo3* in neurogenesis, we found that *Foxo3* shares common targets with the pro-neuronal transcription factor *Ascl1*, an important pro-neural factor regulated by Notch signaling, including Hes1 oscillations. Additionally, *Foxo3* inhibits Ascl1-dependent neurogenesis in normal neural progenitors and neurogenesis in vivo [13,14]. The classical pro-neural factors *Ascl1* and *Neurog2* contribute to neuronal subtype identities by establishing distinct chromatin landscapes and regulating the induction of cell cycle exit and the differentiation of neuro-progenitor cells in the embryonic cortex [15,16]. *Ascl1* and *Neurog2* are crucial for acquiring neuronal identity during nervous system development and are widely used for direct neuronal reprogramming [17]. *Ascl1* is required and sufficient to promote the differentiation of a subset of Cajal–Retzius (CR) cells found in the neocortex and hippocampus during early brain development. CR cells control the migration of glutamatergic neurons and the formation of cortical layers through the secretion of the glycoprotein reelin [18]. Furthermore, it has been suggested that CR cells are involved in a novel excitatory loop of the postnatal hippocampal formation, which potentially contributes to shaping the flow of information between the hippocampus, parahippocampal regions, and entorhinal cortex [19,20].

Our previous studies reported that acute exposure to VPA affects the expression of *Foxo3* and Notch signaling genes in vitro in the SH-SY5Y cell line [21]. Additionally, sex-related differences have been observed in *Foxo3* expression in the hippocampi of acute VPA-exposed rats [22]. With the existence of transcription factors that regulate the expression of *Foxo3*, which have been proposed to be too diverse and are not yet well known for specific regulation, it has been suggested that epigenetic modifications affect FoxO gene expression [23,24]. It has been reported that treatment with trichostatin A (an HDAC inhibitor) or 5-Aza (a DNA methyltransferase inhibitor) induces *Foxo3* expression in mouse embryonic fibroblast cells [25].

In this study, we aimed to observe *Foxo3* expression changes in the hippocampus of the neonatal VPA-exposed animal model, with correlations between *Foxo3* expression and social behaviors. In addition, we studied the epigenetic changes in long-term *Ascl1* and *Foxo3* expression with sex-related differences.

## 2. Results

### 2.1. Neonatal VPA Exposure Upregulated mRNA Expression of the Foxo3 Gene in the 4-Week-Old Rat Hippocampus with Sex-Related Differences

**Figure 1:**
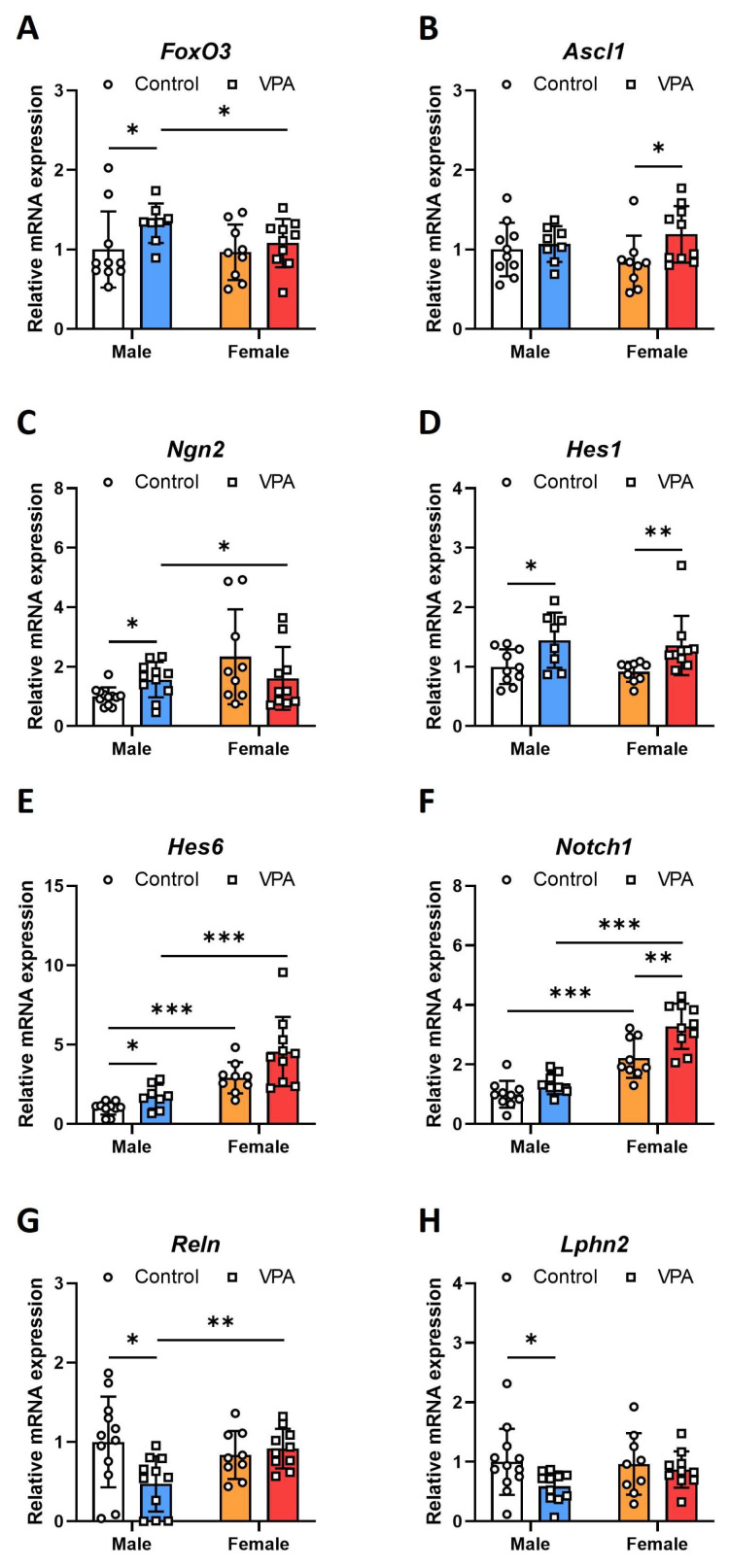
Neonatal VPA exposure upregulates mRNA expression of the *Foxo3* gene in the 4-week-old rat hippocampus with sex-related differences. Gene expressions of *Foxo3* (**A**), *Ascl1* (**B**), *Ngn2* (**C**), *Hes1* (**D**), *Hes6* (**E**), *Notch1* (**F**), *Reln* (**G**), and *Lphn2* (**H**) had sex-related differences in neonatal VPA-exposed hippocampus. Relative mRNA expression measured by RT-qPCR in neonatal VPA-exposed 4-week-old rat hippocampus. Values represent the mean ± standard deviation (**A**–**F**), male control *n* = 10, male VPA *n* = 8, female control *n* = 9, female VPA *n* = 10; (**G**,**H**), male control *n* = 12, male VPA *n* = 11, female control *n* = 9, female VPA *n* = 10). The significant level between the groups is indicated by * *p* < 0.05, ** *p* < 0.01, and *** *p* < 0.001.

Reverse transcription (RT)-PCR was performed to investigate alterations in the mRNA expression of *Foxo3* and related genes in hippocampi after early neonatal VPA exposure. The mRNA expression levels of *Foxo3*, pro-neural factor-related genes, and Notch signaling-related genes in 4-week-old rats after early VPA exposure showed sex-related differences. In 4-week-old male rats, the VPA-exposed group revealed increasing mRNA expression of *Foxo3* (Figure 1A; Mann–Whitney U *p* = 0.033). We also evaluated the changes in the mRNA expression of *Foxo3*-related pro-neural factor genes. Regarding the mRNA expression of *Ascl1* and genes related to Notch signaling, *Hes1* (Figure 1D; t(16) = −2.480, *p* = 0.025), and *Hes6* (Figure 1E; Mann–Whitney U, *p* = 0.033) were increased in the hippocampi of male rats; in contrast, *Ascl1* (Figure 1B; t(17) = −2.222, *p* = 0.040), *Hes1* (Figure 1D; Mann–Whitney U, *p* = 0.003), and *Notch1* (Figure 1F; t(17) = −3.241, *p* = 0.005) were significantly increased in the hippocampi of female rats. In the hippocampi of male rats, the relative mRNA expression of *Ngn2* (Figure 1C; t(8.429) = −2.475, *p* = 0.005) increased. in contrast, that of *Reln* (Figure 1G; t(21) = 2.640, *p* = 0.010) and *Lphn2* (Figure 1H; t(21) = 2.268, *p* = 0.050) decreased. No changes were observed in the female hippocampus. For *Ngn2*, there were significant differences in expression based on sex (F = 5.355, *p* = 0.026) and a significant interaction between sex and treatment (F = 4.694, *p* = 0.037), suggesting that both sex and its interaction with treatment modulate Ngn2 expression in this animal model. *Reln* did not display significant sex-related differences in expression (*p* = 0.269) but did exhibit significant changes due to the interaction between sex and treatment (F = 5.966, *p* = 0.019), highlighting that this gene’s expression is more dependent on the combined effects of sex and treatment rather than on either factor alone (Table S1).

### 2.2. Hippocampal mRNA Expression of Foxo3 Shows a Negative Correlation with Sociality Index in the 4-Week-Old Male Rats

The sociality index was reduced in 4-week-old rats after neonatal VPA exposure, without sex-related differences (Figure S1A; male, Mann–Whitney U, *p* < 0.001; female, t(33) = 4.459, *p* < 0.001). This sociality index decline was maintained at 8 weeks after neonatal VPA exposure (Figure S1B; male, Mann–Whitney U, *p* = 0.005; female, Mann–Whitney U, *p* = 0.040). Males showed a decreasing trend; however, this difference was insignificant (Figure S1C, Mann–Whitney U, *p* = 0.098). Social preference indices observed 8 weeks after neonatal VPA exposure recovered in women and significantly decreased in men (Figure S1D; men, Mann–Whitney U, *p* = 0.011; women, Mann–Whitney U, *p* = 0.715).

**Figure 2:**
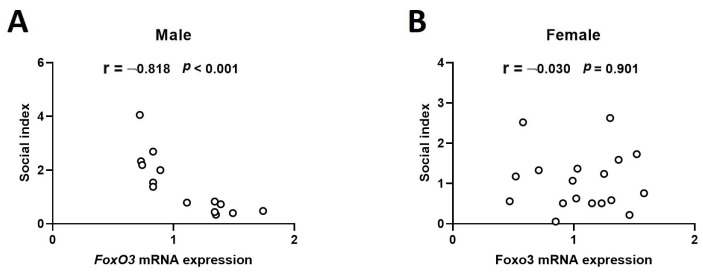
Hippocampal mRNA expression of *Foxo3* shows a negative correlation with the sociality index in the 4-week-old male rat. Sociality was measured by behavioral test in 4-week-old rats exposed to VPA as neonates ((**A**) male *n* = 14; (**B**) female *n* = 18). The correlation between the *Foxo3* mRNA expression measured in the rat hippocampus and sociality index was analyzed by the SPSS v20 program.

In the correlation analysis results between the social index and *Foxo3* mRNA expression, the social index value was negatively correlated with *Foxo3* mRNA expression in the 4-week-old male hippocampus (Figure 2A; Pearson Correlation = −0.818, *p* < 0.001). However, there was no correlation in the correlation analysis for the female sample (Figure 2B; Pearson Correlation = −0.030, *p* = 0.901). In the additional correlation analysis with whole samples, males and females, a significant negative correlation was still observed (Pearson Correlation = −0.423, *p* < 0.016).

### 2.3. Neonatal VPA Exposure Had Long-Term Effects on Foxo3/Ascl1/Notch Protein Expression and Autophagy Signaling in the 4-Week-Old Male Hippocampus

**Figure 3:**
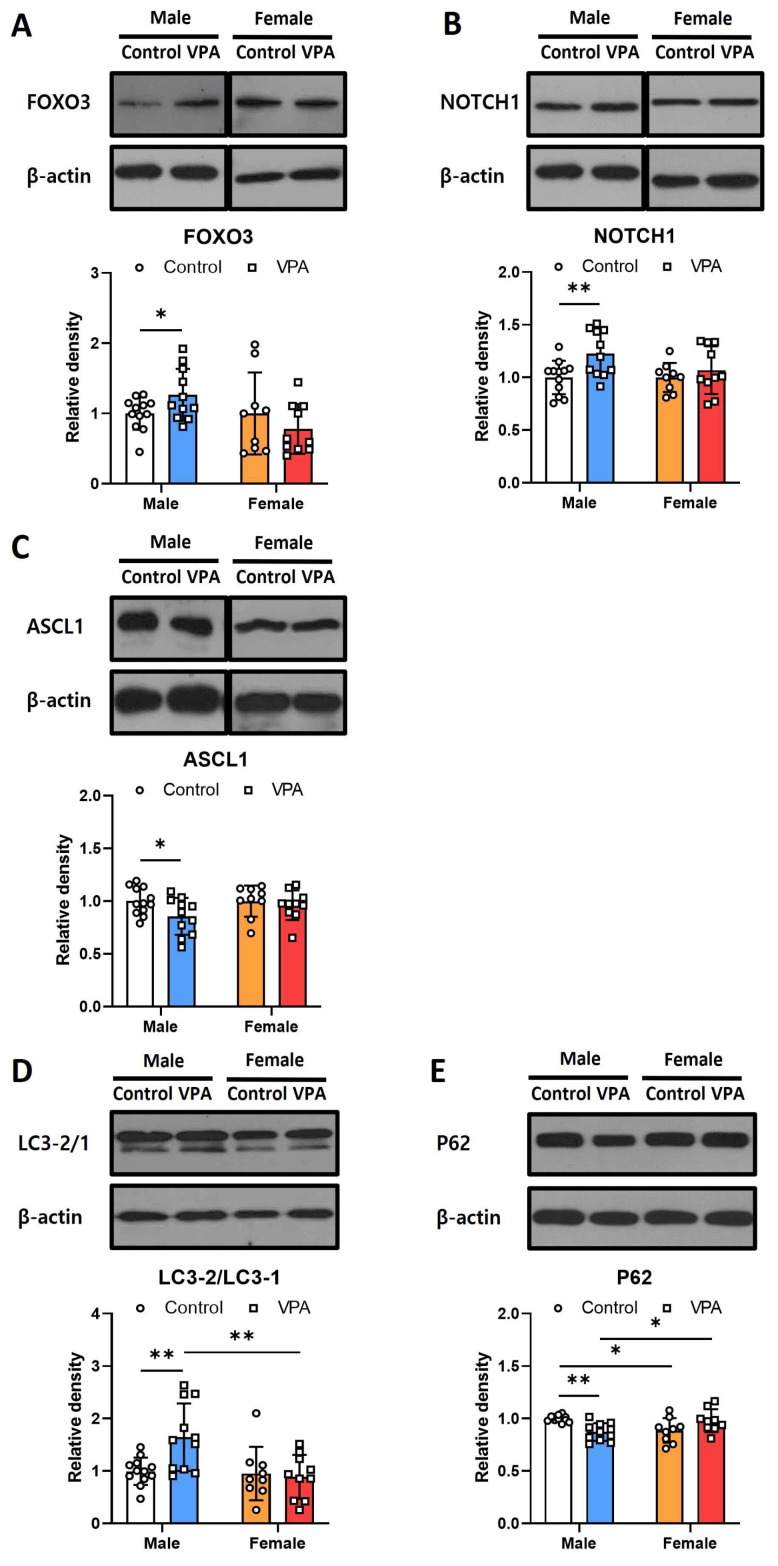
Neonatal VPA exposure affected long-term hippocampal autophagy and Notch signaling in the 4-week-old rat with sex-related differences. Protein levels of FOXO3 (**A**), NOTCH1 (**B**), ASCL1 (**C**), LC3 (**D**), and P62 (**E**) were measured by Western blot in the neonatal VPA-exposed 4-week-old rat hippocampus. Values represent the mean ± Standard deviation ((**A**,**B**,**D**,**E**), male control *n* = 12, male VPA *n* = 11, female control *n* = 8, female VPA *n* = 8; (**C**), male control *n* = 12, male VPA *n* = 11, female control *n* = 12, female VPA *n* = 12). The significant level between the groups is indicated by * *p* < 0.05 and ** *p* < 0.01.

To assess alterations in the protein levels of Foxo3 and Notch signaling-related genes in the male and female hippocampi following early neonatal VPA exposure, we conducted Western blotting analysis using 4-week-old rats. We observed statistically significant increases in the protein levels of Foxo3 (Figure 3A; t(21) = −2.090, *p* = 0.049) and Notch1 (Figure 3B; t(21) = −2.882, *p* = 0.004) exclusively in male rats. Conversely, Ascl1 protein levels were significantly decreased in males at 4 weeks old (Figure 3C; t(21) = 2.260, *p* = 0.035). No alterations were detected in the female samples, indicating sex-related differences in protein expression. In addition, the decrease in Ascl1 protein expression observed in the hippocampal tissue of 8-week-old males remained consistent (Figure S2).

Changes in autophagy-related proteins in the hippocampi of the 4-week-old rats following early VPA exposure were observed by Western blotting. In the hippocampi of the male rats exposed to VPA, the ratio of LC3-2 to LC3-1 increased (Figure 3D; t(12.963) = −3.118, *p* = 0.008), P62 decreased (Figure 3E; t(12.329) = 4.815, *p* < 0.001), and autophagy was activated.

For LC3-2/1, the analysis revealed a significant effect of sex on protein expression levels (F = 7.368, *p* = 0.01) and a significant interaction between sex and treatment (F = 5.831, *p* = 0.021), indicating that sex differentially modulates LC3-2/1 expression in response to treatment. Remarkably, the interaction between sex and treatment exhibited a very significant effect (F = 15.91, *p* < 0.001), suggesting that the expression of P62 is strongly influenced by the combined effects of sex and treatment, highlighting potential sex-specific responses to the treatment conditions (Table S1).

### 2.4. Neonatal VPA Exposure Led to Increased H3K27ac Levels at the Foxo3 Promoter in the Hippocampi of 4-Week-Old Male Rats

**Figure 4:**
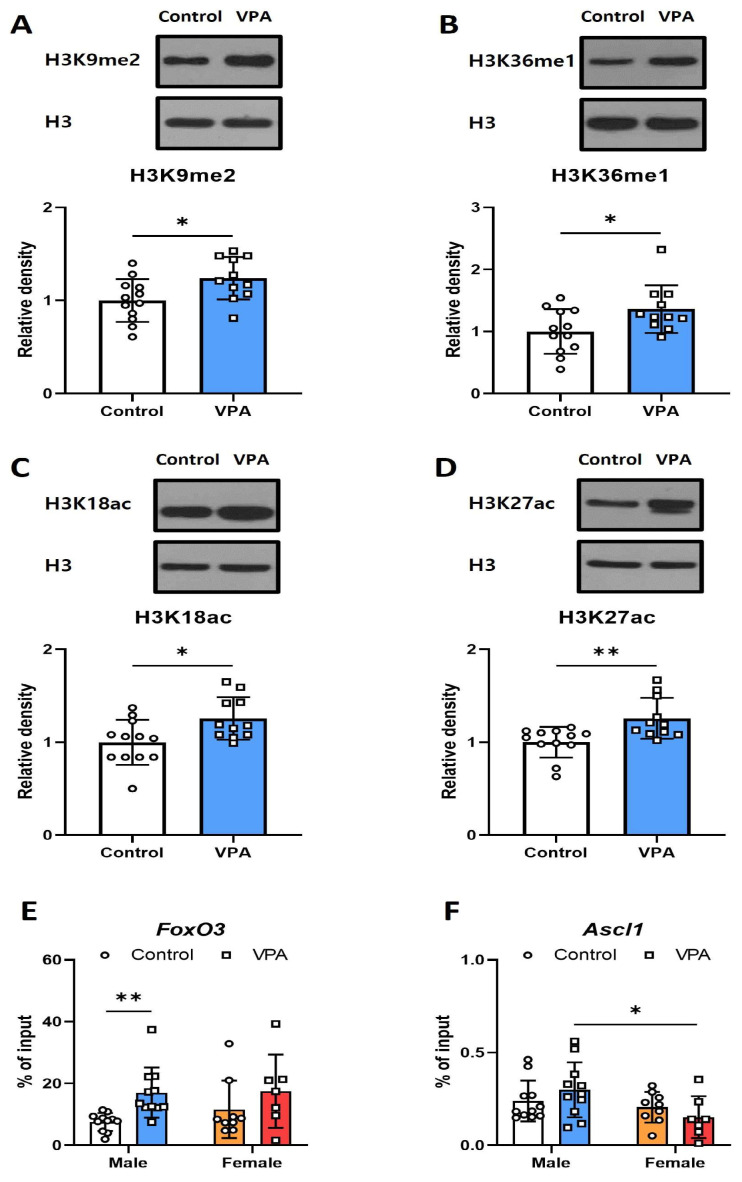
Increased H3K27ac increases *Foxo3* expression in the neonatal VPA-exposed 4-week-old male hippocampus. (**A**–**D**) Histone H3 modification in neonatal VPA-exposed 4-week-old male hippocampus was measured by Western blot. H3K27 acetylation of the promoter regions of *Foxo3* (**E**) and *Ascl1* (**F**) genes was assessed by ChIP-qPCR in neonatal VPA-exposed 4-week-old rat hippocampus. Values represent the mean ± Standard deviation ((**A**–**D**), control *n* = 12, VPA *n* = 11; (**E**,**F**), male control *n* = 11, male VPA *n* = 11, female control *n* = 9, female VPA *n* = 7). The significant level between the groups is indicated by * *p* < 0.05 and ** *p* < 0.01.

Western blot analysis confirmed increased levels of H3K9me2 (Figure 4A; t(21) = −2.484, *p* = 0.020), H3K36me1 (Figure 4B; t(21) = −2.314, *p* = 0.030), H3K18ac (Figure 4C; t(21) = −2.614, *p* = 0.016), and H3K27ac (Figure 4D; Mann–Whitney U *p* = 0.005) in VPA-exposed male neonates. The impact of H3K27 acetylation on the promoter regions of *Foxo3* and *Ascl1* was investigated using ChIP-qPCR with anti-H3K27Ac antibodies. The ChIP-qPCR results revealed a significant increase in H3K27ac at the *Foxo3* promoter in the hippocampus of four-week-old male rats exposed to VPA (Figure 4E; t(20) = −3.652, *p* = 0.002); however, this increase was not observed in females. Additionally, females exhibited a decreased association of H3K27Ac with the promoter region of *Ascl1* compared to males in the VPA-treated groups (Figure 4F; t(16) = 2.248, *p* = 0.042).

For *Foxo3*, the data indicate a significant response to treatment (F = 8.078, *p* = 0.008), demonstrating that treatment significantly influences *Foxo3* binding independently of sex, as neither the sex effect nor the interaction between sex and treatment were significant. In contrast, *Ascl1* showed significant differences in response to sex (F = 5.563, *p* = 0.024), suggesting a sex-dependent variation in *Ascl1* binding (Table S1).

### 2.5. Neonatal VPA Exposure Decreased DNA Methylation at the Promoter Regions of Foxo3 and Ascl1 in the 4-Week-Old Rats’ Hippocampi with Sex-Related Differences

**Figure 5:**
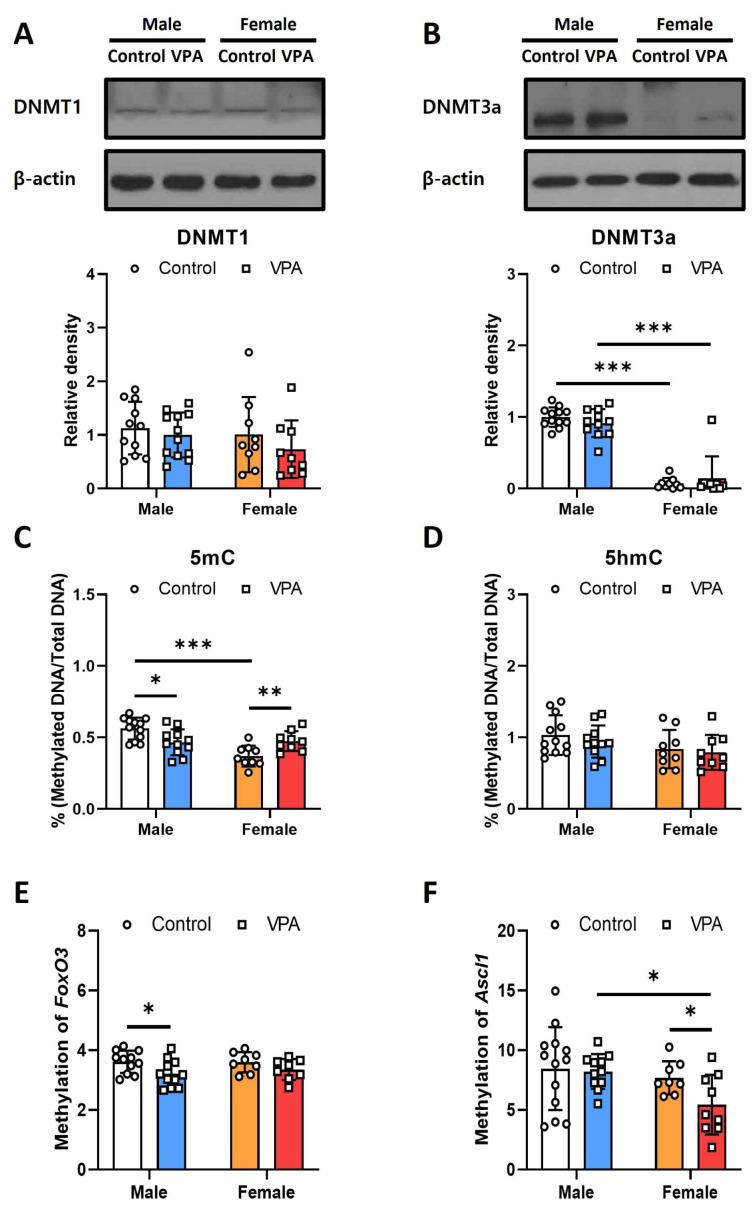
Neonatal VPA exposure decreased DNA methylation at the promoter regions of Foxo3 and Ascl1 in the 4-week-old rat hippocampus with sex-related differences. (**A**,**B**) Expression level of DNA methylation-related proteins were measured by Western blot in neonatal VPA-exposed rat hippocampus. (**C**,**D**) Percentage of DNA methylation in neonatal VPA-exposed rat hippocampus was assessed by Global DNA methylation and hydroxymethylation assay. (**E**,**F**) Methylation of the Foxo3 and Ascl1 genes promoter regions was assessed by the MSRE-based qPCR method. Values represent the mean ± Standard deviation (**A**–**E**), male control *n* = 12, male VPA *n* = 11, female control *n* = 8, female VPA *n* = 8; (**F**), male control *n* = 13, male VPA *n* = 11, female control *n* = 8, female VPA *n* = 9). The significance level between the groups is indicated by * *p* < 0.05, ** *p* < 0.01 and *** *p* < 0.001.

Western blotting and ELISA were performed to investigate alterations in DNA methylation in the male and female rats’ hippocampi following neonatal VPA exposure. The protein levels of the methylation-related genes *Dnmt1* and *Dnmt3a* were not different between the control and VPA groups (Figure 5A,B). *DMNT3A* expression significantly differed between the males and females (Figure 5B; control, t(18) = 17.162, *p* < 0.001; VPA, Mann–Whiney U, *p* < 0.001).

In the ELISA analysis, 5mC levels were significantly increased in males (Figure 5C; t(21) = 2.744, *p* = 0.012) and females (Figure 5C; t(16) = −3.114, *p* = 0.044). Sex-related differences were also observed in the control group (Figure 5C; t(19) = 5.775 *p* < 0.001); however, there were no differences in 5-hmC levels (Figure 5D). For 5-mc, there were significant effects of sex (F = 13.811, *p* = 0.001) and a very significant interaction between sex and treatment (F = 16.402, *p* < 0.001), suggesting that both sex and the interaction with treatment are critical determinants of 5-mc methylation levels. Conversely, while 5-hmc showed moderate sex-dependent variation (F = 4.67, *p* = 0.037), neither treatment alone nor the interaction between sex and treatment significantly influenced its methylation levels (Table S1).

Neonatal VPA exposure affected DNA methylation levels with sex-related differences. The methylation level of the *Foxo3* promoter region was reduced only in the VPA-exposed male hippocampus (Figure 5E; t(21) = 2.441, *p* = 0.024), and that of the *Ascl1* promoter region was decreased only in the VPA-exposed female hippocampus (Figure 5F; t(12.759) = 2.318, *p* = 0.038). The treatment significantly influenced the methylation levels of *Foxo3* (F = 7.035, *p* = 0.012), indicating a responsive alteration in methylation due to external factors, although no significant effects were observed from sex or the interaction between sex and treatment. In contrast, *Ascl1* showed significant variation in methylation related to sex (F = 5.004, *p* = 0.031), while treatment alone and the interaction between sex and treatment did not significantly affect methylation levels, highlighting sex-specific methylation patterns for *Ascl1* (Table S1).

## 3. Discussion

ASD comprises several heterogeneous neurodevelopmental defects. Prenatal VPA exposure has been reported to impair postnatal cognitive function and sociality and cause changes in embryonic and adult neurogenesis and adolescent/adult neurobehavioral phenotypes, creating a well-known animal model for ASD [26]. It has been suggested that postnatal cognitive dysfunction is likely correlated with the untimely enhancement of embryonic neurogenesis, which leads to the depletion of the neural precursor cell pool and, consequently, a decreased level of adult neurogenesis in the hippocampus. Moreover, hippocampal neurons in the offspring of VPA-treated rats show abnormal morphology and activity [27].

Several studies have suggested that the abnormal transmission of neural signaling pathways is associated with the pathogenesis of ASD. FOXOs are multitasking proteins that are key regulators of the cell cycle, apoptosis, metabolism, and oxidative stress responses [28]. It has been suggested that FOXOs are required for the maintenance of neural stem/progenitor cell (NSPC) quiescence and clearance of reactive oxygen species (ROS) in neural stem cells of the brain [29,30]. It has also been suggested that progressively increasing FOXO expression in aging human and mouse brains protects against degeneration [31]. FOXO-dependent cell-protective mechanisms, which induce the expression of ROS-detoxifying enzymes and autophagy, may be important for determining cell fate [32,33]. Thus, *Foxo3* may play an important role in mitochondrial function and cell survival under oxidative stress by interacting with mitochondrial biogenesis-related genes [34]. It was also observed that *Foxo3* directly regulates a network of autophagy genes in adult NSPCs [10].

Autophagy is a conserved intracellular lysosomal degradation pathway that plays a key role in regulating developmental pathways. Autophagy affects stem cell differentiation by degrading *Notch1* [35]. Recent studies have led to the recognition of the role of the Notch signaling pathway in early neurodevelopment, learning, memory, and late-life neurodegeneration [36]. Increased expression of Notch signaling pathway-related molecules, such as *Notch1*, has been reported in the prefrontal cortex, hippocampus, and cerebellum following prenatal VPA exposure. Furthermore, (3,5-Difluorophenacetyl)-L-alanyl-S-phenylglycine-2-butyl Ester (Dapt), a Notch pathway inhibitor, improves autistic-like behaviors by regulating autophagy and affecting the morphology of dendritic spines [37]. In this study, increased *Notch1* expression was observed in VPA-exposed 4-week-old male rats’ hippocampi. Decreased *Reln* and *Lphn2* mRNA expression associated with CR cells may be affected by decreased *Ascl1* levels in VPA-exposed males [18]. Additionally, increased *Foxo3* may inhibit *Ascl1*-dependent neurogenesis processes [13]. In this study, we also observed increased hippocampal *Foxo3* mRNA expression, which correlated with sociality index values from behavioral tests with VPA-exposed 4-week-old males. *Foxo3* is a transcription factor that influences the generation and differentiation of NSCs [11] and may have the potential to induce or inhibit specific brain cell differentiation, possibly related to neuropsychiatric pathogenesis.

*Foxo3* has been suggested to be an immediate early glucocorticoid receptor target whose transcription is further enhanced by conditions that mimic metabolic stress [23]. Glucocorticoids have also been suggested to regulate *Foxo3* transcription [38]. In previous reports, we observed increased hippocampal *Foxo3* expression in males exposed to acute VPA, with increased cortisol levels in the cerebral cortex [22,39]. Another study has reported increased cortisol levels in the cerebral cortex following neonatal VPA exposure. This may have led to the increase in *Foxo3* expression observed in this study; however, the lack of evidence for changes in hippocampal cortisol levels makes it difficult to establish a correlation [40].

Epigenetic regulation by perinatal environmental factors is an important intrinsic process that interacts with transcription factors and environmental cues to regulate pluripotent NSCs’ differentiation [41]. In this study, H3K27ac-related open chromatin changes and decreased DNA methylation of the *Foxo3* promoter may partially explain the increased *Foxo3* expression observed in the neonatal VPA-exposed male hippocampus. Histone acetylation, which may be induced by HDAC inhibitors such as VPA, is a critical epigenetic modulation that alters chromatin architecture and regulates gene expression through structural changes in chromatin. There are few reports on the changes in the expression or activity of *DNMT* genes by external environmental factors, and it has been suggested that *Dnmt1* and *Dnmt3a* are required for synaptic plasticity, learning, and memory through their overlapping roles in maintaining DNA methylation and modulating neuronal gene expression in adult CNS neurons [42]. This study showed a significant difference in *Dnmt3a* expression according to sex in 4-week-old rats’ hippocampal tissue; however, no change was observed due to neonatal VPA exposure. In addition, various histone modifications, including methylation at *H3K9* and *H3K36*, have been observed in the male hippocampus exposed to neonatal VPA.

The VPA-exposed animal model is one of the most used ASD models which shows sex differences in the results of studies on behavior, endocrine or immune systems, and brain connectivity [43]. The effects of prenatal exposure to VPA on the hippocampal network and neuron excitability in rats have been investigated, revealing age- and sex-dependent variations in excitatory responses and synaptic plasticity, with some changes persisting into older age while others were transient [44]. In this study, we also observed sex differences in long-term epigenetic and protein expression changes in the neonatal VPA-exposed animal model. The epigenetic factors may play a significant role in the sex differences observed in the onset of neurodevelopmental disorders.

## 4. Materials and Methods

### 4.1. Animal Model

Seven-week-old male and female Sprague–Dawley rats were purchased from Samtako (Gyeonggi-do, Republic of Korea). During the 1-week adaptation period, rats were maintained on a standard regular 12 h light–dark cycle at ambient temperature (22 °C ± 2 °C) and humidity (50% ± 10%), with food and water available ad libitum. Subsequently, one male and two female rats were mated. After pregnancy was confirmed, the female rats were placed in their respective cages. Pregnant mice were divided into two groups, and the farrowing date of neonatal pups was considered PND 0 (The litter size 7–15). Pups from the same mothers were subcutaneously injected with 300 mg/kg VPA (Sigma, St. Louis, MO, USA) or normal saline twice daily randomly on PND 2 and 3 and once on PND 4. There were more than three pregnant rats per group, and all rats within the same litter were used in the study. Behavioral tests were performed 4 or 8 weeks after birth, and the hippocampi were isolated after sacrifice following inhalational anesthesia. The samples were stored at −80 °C until the experiment. This study was approved by the Institutional Animal Care and Use Committee of Eulji University (EUIACUC 20-05).

### 4.2. Social Interaction Test

The social interaction test was conducted at 4 weeks of age, following a previously described method [45]. Considering the circadian rhythms, the test was performed between 9:00 a.m. and 12:00 p.m. The test chamber comprised three compartments. The subject was placed in the middle compartment with the doors closed for 5 min before the test. After that, a strange rat (same age and no previous contact) was introduced into the wired cage at the left compartment (stranger zone), while the other wired cage remained empty (empty zone) during the 10 min sociability test. The social index was measured by comparing the time spent around the stranger zone with the time spent in the empty zone. In the social preference test, new rats (the same age with no previous contact) were placed in the empty right compartment. The left compartment was considered the familiar zone, and the right compartment was considered the stranger zone, where a new rat was introduced. The social preference test was conducted for 10 min, and the social preference index was measured by comparing the time spent in the familiar and stranger zones (stranger zone/familiar zone). While the sociality index quantifies the frequency and duration of an animal’s interactions within its natural group setting, the social preference test specifically evaluates an animal’s selective affinity for social versus nonsocial stimuli in a controlled experimental environment.

### 4.3. Real-Time Quantitative Polymerase Chain Reaction

Total RNA was extracted from the hippocampus using the RNeasy Mini Kit (Qiagen, Hilden, Germany). Complementary DNA (cDNA) was synthesized using an iScript^TM^ cDNA synthesis kit (Bio-Rad, Hercules, CA, USA) from 100 ng of total RNA. cDNA was mixed with SYBR Green Super Mix (Bio-Rad, Hercules, CA, USA) and primers and amplified using a CFX96TM Real-Time System (Bio-Rad, Hercules, CA, USA). Primer sequences used to amplify the target genes are listed in Table S2. mRNA expression was normalized to that of GAPDH. Raw data were analyzed using the 2^−ΔΔCT^ method.

### 4.4. Histone Extraction and Western Blot

According to the manufacturer’s instructions, histone proteins were isolated from the hippocampus using a histone extraction kit (Abcam, Cambridge, UK). Total protein was isolated from the hippocampal tissue using RIPA buffer (ATTO, Tokyo, Japan) with proteinase and phosphatase inhibitors (ATTO, Tokyo, Japan). The protein concentration was measured using a BCA assay kit (Thermo Scientific, Waltham, MA, USA). Equal amounts of histone protein (10 µg) or total protein (20 µg) were separated by SDS-PAGE and transferred onto nitrocellulose membranes (Pall, Port Washington, NY, USA). The membranes were blocked with 5% non-fat milk in TBST buffer for 1 h at 4 °C and incubated with primary antibody diluted in TBST at 4 °C overnight. The membranes were incubated with horseradish peroxidase-labeled secondary antibodies diluted in TBST for 1 h after TBST rinse. The list of antibodies is in Table S3. After washing with TBST buffer, the membranes were incubated with West Femto Maximum Sensitivity Substrate (Thermo Scientific, Waltham, MA, USA). Protein expression levels were analyzed using ImageJ lab software https://imagej.net/ij/ (NIH, Bethesda, MD, USA) with exposed X-ray film.

### 4.5. Chromatin Immunoprecipitation-Quantitative Polymerase Chain Reaction (ChIP-qPCR)

According to the manufacturer’s instructions, chromatin was isolated from the hippocampus using a chromatin extraction kit (Abcam, Cambridge, UK). The ChIP assay was performed according to the ChIP kit-One step (Abcam, Cambridge, UK) protocol using an acetyl-H3K27 antibody (Abcam, Cambridge, UK). DNA fragments were assessed by qPCR using primer sequences. The primers were designed based on the gene promoter region. The primer sequences are listed in Table S2.

### 4.6. Global DNA Methylation and Hydroxymethylation Assay

Total genomic DNA was extracted from the hippocampus using a Dneasy Blood and Tissue Kit (Qiagen, Hilden, Germany). For Global DNA methylation (5-methylcytosine/total DNA) and hydroxymethylation (5-hmc/total DNA), 100 ng of extracted DNA was analyzed using the Global DNA Methylation Kit (Abcam, Cambridge, UK) and Global DNA Hydroxymethylation Kit (Abcam, Cambridge, UK) according to the manufacturer’s instructions.

### 4.7. Methylation-Sensitive Restriction Enzymes (MSRE)-Based qPCR Assay

Genomic DNA (1 μg) was diluted in 50 μL rCutsmart buffer^TM^ (Biolabs, Boston, MA, USA) and then divided into three aliquots. Two aliquots were digested with *Msp*I (Biolabs, Boston, MA, USA) or *Hpa*II (Biolabs, Boston, MA, USA), and one aliquot was not digested. The mixtures were incubated at 37 °C for 1 h. SYBR Green supermix (Bio-Rad, Hercules, CA, USA) and CFX96TM Real-Time System (Bio-Rad, Hercules, CA, USA) were used for amplification with primers. Primer sequences used to amplify the target genes are listed in Table S2. Raw data were analyzed using the formula ΔCT (MSPI-UD) − ΔCT (HpaII-D) [46]. 

### 4.8. Statistical Analysis

Data were analyzed using GraphPad Prism 7 (GraphPad Software Inc., La Jolla, CA, USA). All results are expressed as mean ± standard deviation. Statistical analyses were conducted using two-way ANOVA, unpaired *t*-test, Mann–Whitney U, and correlation test using SPSS v20 (IBM, Armonk, NY, USA). We conducted the analysis under the assumption that two-way ANOVA requires normal distribution and homogeneity of variances, thus only performing it when the experimental groups satisfy the normal distribution. Significant *p*-value data were denoted as * *p* < 0.05, ** *p* < 0.01, and *** *p* < 0.001.

## 5. Conclusions

This study provides biological clues to sex-related differences in neurodevelopmental disorders by observing that neonatal VPA exposure induces hippocampal gene expression differently according to sex as a long-term epigenetic mechanism. However, considering the multiple epigenetic changes induced by VPA exposure, studies on simple H3K27 acetylation and single DNA methylation sites in the promoter regions have limitations in interpreting gene expression changes associated with behavioral changes. Further research is needed to determine *Foxo3* and *Ascl1* gene expression regulatory mechanisms and their relationship with neuropsychiatric diseases.

## Data Availability

The authors declare that all required data have been presented in the manuscript. The datasets did not contain any software code needing to be archived.

## Supplementary Materials

The following supporting information can be downloaded at: https://www.mdpi.com/article/10.3390/ijms25105287/s1.
