# Supplementary material for: Long-Term Epigenetic Regulation of Foxo3 Expression in Neonatal Valproate-Exposed Rat Hippocampus with Sex-Related Differences
Source: Int J Mol Sci. 2024 May 13;25(10):5287. doi: 10.3390/ijms25105287 (PMC11121443; doi:10.3390/ijms25105287)
Supplement: Supplementary file 1 [file ijms-25-05287-s001.zip › ijms-2939642-supplementary.pdf]

Supplementary data

Figure S1. Sociability test.

(A), (C) After 4 and 8 weeks of neonatal VPA exposure, the social index was measured by comparing the time spent in the empty zone and the stranger zone through a social interaction test.
(B), (D) The social preference index was measured by comparing the time spent in the stranger zone and the familiar zone. Values represent the mean ± Standard deviation.
The significance level between the groups is indicated by $* p < 0.05$, $** \ p < 0.01$ and $*** p < 0.001$.
((A), male control n = 21, male VPA n = 22, female control n = 20, female VPA n = 15;
(B), male control n = 5, male VPA n = 6, female control n = 4, female VPA n = 6;)
(C), male control n = 19, male VPA n = 20, female control n = 16, female VPA n = 15;
(D), male control n = 4, male VPA n = 6, female control n = 5, female VPA n = 6)).

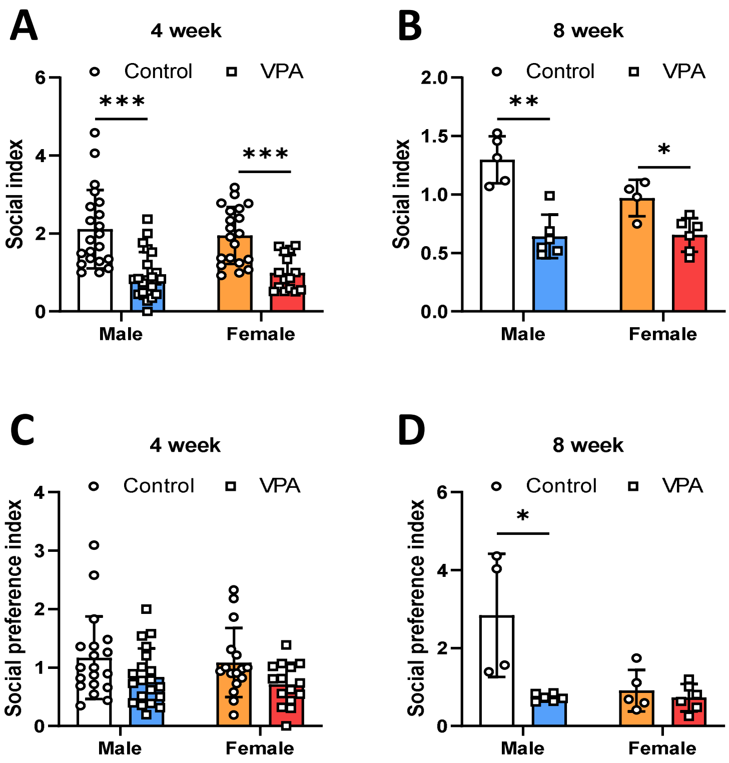

Figure S2. Neonatal VPA exposure have long-term effects on FOXO3 and Notch signaling protein expression in the 4 and 8 weeks old male hippocampus.

The protein levels of *Foxo3* (Fig. 3A; $t(21) = -2.090$, $p = 0.048$) and *Notch1* (Fig. 3C; $t(21) = -2.882$, $p = 0.009$, $p = 0.009$) were significantly increased only in the 4 weeks old male rats after VPA exposure. *Ascl1* protein levels were significantly decreased in 4 weeks old males (Fig. 3B; $t(21) = 2.260$, $p = 0.026$) and 8 weeks old males (Fig. 3B; $t(14) = 2.334$, $p = 0.035$).

No changes were observed in the females, and no sex-related differences were observed.

The significance level between the groups is indicated by $* p < 0.05$, $** \ p < 0.01$ and $*** p < 0.001$.

((A) 4 weeks control n = 12, 4 weeks VPA n = 11, 8 weeks control n = 8, 8 weeks VPA n = 8;

(B) 4 weeks control n = 12, 4 weeks VPA n = 11, 8 weeks control n = 12, 8 weeks VPA n = 12;

(C) 4 weeks control n = 12, 4 weeks VPA n = 11, 8 weeks control n = 8, 8 weeks VPA n = 8).

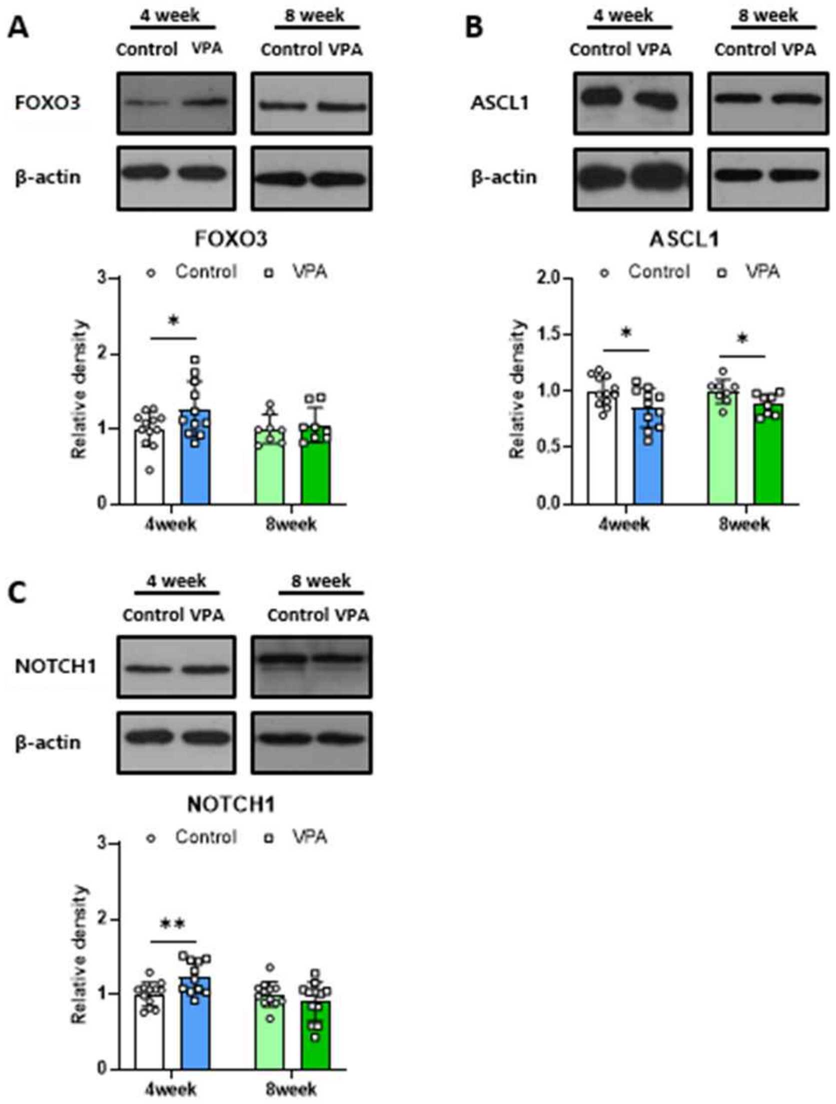

Table S1. Two-way ANOVA results Summary for VPA exposure and sex differences

Figure	Test	Gene	Sex		Treatment		Sex & Treatment	
			F	*p*-value	F	*p*-value	F	*p*-value
1	qPCR	*Ngn2*	5.355	0.026	0.074	0.787	4.694	0.037
		Notch1	64.787	0.000	13.525	0.001	3.249	0.081
		Reln	1.260	0.269	3.225	0.080	5.966	0.019
		Lphn2	0.874	0.356	3.636	0.064	1.441	0.237
3	Western blot	LC3-2/1	7.368	0.010	3.952	0.054	5.831	0.021
		P62	0.007	0.933	0.433	0.515	15.910	0.001
4	ChIP-qPCR	*Foxo3*	0.714	0.404	8.078	0.008	0.453	0.506
		Ascl1	5.563	0.024	0.007	0.936	2.298	0.139
5	DNA methylation & hydroxymethylation assay	5-mc	13.811	0.001	0.029	0.866	16.402	0.000
		5-hmc	4.670	0.037	0.711	0.405	0.080	0.778
	MERE-based qPCR	*Foxo3*	0.138	0.713	7.035	0.012	0.471	0.497
		Ascl1	5.004	0.031	2.472	0.124	1.613	0.212

Table S2. Primer list

Primers for RT-qPCR

Gene name	Forward sequence (5'→3'	Reverse sequence (5'→3')
Gapdh	AGTGCCAGCCTCGTCTCATA	AGAGAAGGCAGCCCTGGTAA
FoxO3	CAGACCCTCAAACTGACCGAA	GCTGCTAACAGTCTCTGCTGG
Ascl1	GGGATCCTACGACCCCCTTAG	AGACACAGGATCTCCTGCCAT
Ngn2	CGGGTAGGATGTTCGTCAAGT	ACCCAGCAGCATCAGTACCT
Hes1	GCGCCGGGCAAGAATAAA	TTCCAGAATGTCTGCCTTCTCC
Hes6	AGG CCA AGC TAG AGA ACG C	GGATGTAGCCAGCAGCGAA
Notch1	GTGCGCTCTAGGTGCCAA	TCT GAC ATG GGT TGG AGA TGC
Reln	AGTCCCTGGGGTTCTACCTC	CATGCTCGAGGCAAGTTTCG
Lphn2	TTTTCCCCTGCCAGAGAGAT	CTGTGGAAGCCATGCAGAGA

Primers for ChIP-qPCR

Gene name	Forward sequence (5'→3'	Reverse sequence (5'→3')
FoxO3	AACCGGAAAAGCGTAGCTCA	GGTATAGGGTTGTCGCGGAG
Ascl1	TTTTCCAAGTTCTCAAGAGACTCC	GGTTTTAAAAGAGGAAAGGGAAAA

Primers for MSRE-based qPCR

Gene name	Forward sequence (5'→3'	Reverse sequence (5'→3')
FoxO3	GAGGTGTATTTTCGCGGGGA	AGGTGATGAACGTGCTGGTC
Ascl1	CAGGTTAACCAACTTGACCCG	GAACTGATGCGCTGCAAACG

Table S3. Antibody list

Antibody	Dilution for WB	Manufacturer	Cat. No.
β-actin	1:3000	Cell signaling	4970
FOXO3	1:1000	Cell signaling	12829
ASCL1	1:1000	Abcam	ab74065
NOTCH1	1:1000	Cell signaling	3608
LC3	1:1000	Cell signaling	4108
P62	1:1000	Abcam	ab56416
H3K9me2	1:1000	Abcam	ab1220
H3K36me1	1:1000	Cell signaling	14111
H3K18ac	1:1000	Abcam	ab1191
H3K27ac	1:1000	Abcam	ab4729
HistoneH3	1:1000	Abcam	ab1791
DNMT1	1:1000	Abcam	ab13537
DNMT3a	1:1000	Abcam	ab2850
HRP-labeled rabbit	1:5000	Invitrogen	31460
HRP-labeled mouse	1:5000	Invitrogen	31430
